# Efficacy and safety of Shengjiang Xiexin decoction on irinotecan-induced diarrhea in small cell lung cancer patients: a multicenter, randomized, double-blind, placebo-controlled trial

**DOI:** 10.1186/s13020-024-01025-6

**Published:** 2024-11-04

**Authors:** Chao Deng, Qing Liu, Meng Yang, Hui-juan Cui, Yang Ge, Qin Li, Shi-jie Zhu, Guo-wang Yang, Zhi-guo Zhang, Yu Gao, Yan-ni Lou, Li-qun Jia

**Affiliations:** 1 Department of Medical Oncology, Integrated Traditional Chinese and Western Medicine, China-Japan Friendship Hospital, No.2, East Street, Ying Hua Yuan, Chao Yang District, Beijing, China; 2 Department of Respiratory and Critical Care Medicine, China-Japan Friendship Hospital, Beijing, China; 3 Department of Medical Oncology, Beijing Chao-Yang Hospital, Capital Medical University, Beijing, China; 4 Department of Medical Oncology, Beijing Friendship Hospital, Capital Medical University, Beijing, China; 5 Department of Medical Oncology, Wangjing Hospital of China Academy of Traditional Chinese Medicine, Beijing, China; 6 Department of Medical Oncology, Beijing Hospital of Traditional Chinese Medicine, Capital Medical University, Beijing, China; 7 Department of Medical Oncology, Beijing Daxing District People’s Hospital, Beijing, China; 8 Department of Traditional Chinese Medicine, Peking University Third Hospital, Beijing, China

**Keywords:** Irinotecan-induced diarrhea, Randomized controlled trial, Shengjiang Xiexin decoction

## Abstract

**Background:**

Irinotecan is a standard chemotherapeutic agent in small cell lung cancer (SCLC), however, as a common adverse reaction, diarrhea limits the use of irinotecan. Shengjiang Xiexin decoction (SXD) has been used in various gastrointestinal diseases in China two thousand years ago. We designed this clinical trial to supply more evidences on the use of SXD as prophylaxis for irinotecan-induced diarrhea, especially for high-risk population predicted by gene testing of uridine diphosphate glucuronosyl transferase 1A1 (UGT1A1).

**Methods:**

In this clinical trial, 120 patients with SCLC were recruited from six hospitals in China. They received two cycles of chemotherapy, meanwhile they were randomized to receive SXD or placebo for 14 days of oral administration in each cycle of chemotherapy. The primary outcome is the incidence of diarrhea. And secondary outcomes include the the degree of diarrhea and neutropenia, the number of chemotherapy cycles with diarrhea, first occurrence time and duration of diarrhea. To evaluate the effect of SXD on the intestine, a rat model with delayed-onset diarrhea induced by irinotecan was established, and the expression of inflammatory factors including IL-1β, IL-6 and TNF-α, anti-inflammatory factors including IL-10, TGF- β in jejunal tissue was detected by ELISA.

**Results:**

101 patients (53 in SXD group, 48 in placebo group) completed the trial. The incidence of diarrhea in SXD group and placebo group were 26.42% (14/53) and 52.08% (25/48), respectively (P < 0.05), and the degree of diarrhea also had significant differences (P < 0.05). In UGT1A1 high-risk population, the incidence of diarrhea in two groups were 9.09% and 66.67% (P < 0.05), but there was no significant differences in UGT1A1 low-risk population. The incidence of neutropenia with degree 1–3 between two groups was 20.75% vs 20.83%, 13.21% vs 18.57%, 9.43% vs 20.83% (P < 0.05). No severe adverse events were reported in any group. And animal studies had shown SXD reduced content of IL-1β, IL-6, TNF-α, increased content of IL-10, TGF-β in jejunum tissue.

**Conclusions:**

SXD had a prophylactic effect in the diarrhea induced by irinotecan, especially for UGT1A1 high-risk population, and this effect from SXD appeared to be maintained the completion of chemotherapy schedule. The mechanism of action of SXD was related to the regulation of inflammatory factors.

*Trial registration* Chinese Clinical Trial Register: ChiCTR1800018490. Registered on 20 September 2018. https://www.chictr.org.cn/showproj.html?proj=25250. The preliminary protocol of this clinical study has been published in the journal “Trials” in the form of protocol before this paper (Deng et al. in Trials 21:370, 2020).

**Supplementary Information:**

The online version contains supplementary material available at 10.1186/s13020-024-01025-6.

## Introduction

Irinotecan is a commonly used chemotherapeutic drug in the treatment of small cell lung cancer, and plays an anticancer role by inhibiting deoxyribonucleic acid (DNA) replication and ribonucleic acid (RNA) synthesis of tumor cells [1, 2]. In the liver, irinotecan is converted into its active form 7-ethyl-10-hydroxy-camptothecin(SN-38), irinotecan and SN-38 are excreted into the circulation for anti-tumor effect, meanwhile SN-38 is also transformed into deactivated form SN-38 glucuronide (SN-38G) by uridine diphosphate glucuronosyl transferases (UGTs). Irinotecan and its metabolites were released into the intestine via bile, SN-38G reconverts into SN-38 by β-glucosidase in the intestine, causing intestinal mucosal damage and delayed diarrhea, which is the main adverse reaction of irinotecan [3, 4].

Delayed diarrhea is defined as diarrhea occurring more than 24 h after administration of irinotecan [5]. 50–80% of patients accepting irinotecan alone or in combination appear all grades of diarrhea, and 11–32% of patients are complicated by diarrhea above grade 3 [5–7].

The occurrence of diarrhea seriously affects the efficacy of its clinical application. According to the relevant consensus by the Canadian Working Group on chemotherapy induced diarrhea, the patient with grade 1–2 diarrhea should accept dietary management and standard-dose loperamide, if the diarrhea is not resolved, loperamide is needed to upgrade to high-dose, and if the diarrhea is progressed to grade 3–4, octreotide, antibiotics or replenishment of water and electrolytes are recommend to use in the hospital [6]. However, the effect of loperamide is limited and with a risk of cardiac arrhythmia [8], and octreotide also has dubious curative effect [9].

It is currently recognized that uridine diphosphate glucuronosyl transferase 1A1 (UGT1A1) gene has a predictive effect on the risk of irinotecan diarrhea, and the application of irinotecan should be adjusted individually according to UGT1A1 genotype [10, 11]. For the balance of anti-tumor efficacy and adverse effect, the Dutch Pharmacogenetics Working Group (DPWG) recommends a 70% starting dose in mutant-type UGT1A1 patients, and no dose reduction in wild-type patients [12]. In addition, physical condition of patients is also a crucial consideration in clinical practice.

Several classic Traditional Chinese medicine (TCM) prescriptions have shown efficacy in treating or preventing irinotecan-induced diarrhea [13]. In a small sample observational study, Chinese patients with recurrent small cell lung cancer (SCLC) accepting irinotecan-based chemotherapy were enrolled, if delayed diarrhea occurred in the first cycle of chemotherapy, Banxia Xiexin decoction will be orally administered before the second cycle, and 4 of 5 patients experienced relief from diarrhea symptoms using Banxia Xiexin decoction [14]. Another randomized controlled trial involving 41 patients treated with irinotecan. Banxia Xiexin decoction markedly reduced frequency of severe grade 3 or 4 diarrhea as compared to the blank control group, but there was no difference in frequency and duration of diarrhea [15]. Another Chinese herbal compound, Huangqin decoction (PHY906) exhibited similar efficacy. A phase I, double-blind, randomized, placebo-controlled, crossover study of PHY906 involved 17 patients with irinotecan-based chemotherapy, PHY906 decreased the overall incidence of grade 3 or 4 diarrhea and the use of antidiarrheal drugs loperamide and lomotil [16].

As another classical prescription of TCM, SXD has been used in the treatment of gastrointestinal disharmony for more than 2000 years [17]. In a single-arm cohort study, SXD reduced the incidence of irinotecan-induced diarrhea, especially in the high-risk population with UGT1A1 gene mutation [18]. Furthermore, mechanism of action of SXD involve intestinal cells apoptosis, intestinal stem cells proliferation, and pharmacokinetics of irinotecan and its metabolites [19, 20].

At present, it is lack of high-quality clinical evidence to support herbal medicines to guard against irinotecan-induced diarrhea. Therefore, we conducted this randomized controlled clinical trial to evaluate the efficacy and safety of SXD in the prevention of irinotecan-induced diarrhea, and stratified analysis based on UGT1A1 genotype was performed by comparing its efficacy in different risk groups of diarrhea, to verify the efficacy advantage in high-risk population.

## Methods

### Study design

The present study was designed as a multicenter, randomized, double-blind, placebo-controlled study, and was registered in the Chinese Clinical Trial Registry (ChiCTR) on 20 September 2018. Registration number was ChiCTR2400085857.

Participants were recruited from six hospitals in Beijing, China, including China-Japan Friendship Hospital (Ethical Approval Document No. 2018-82-K57), Beijing Friendship Hospital Affiliated with Capital Medical University (2019-P2-016-02), Beijing Hospital of TCM Affiliated with Capital Medical University (2018BL-057-02), Wangjing Hospital of China Academy of Traditional Chinese Medicine (WJEC-KT-2018-045-P001), Beijing Chaoyang Hospital Affiliated with Capital Medical University (2018-KE-313) and Beijing Daxing District People's Hospital (20181231LLKYLX-2-11).

### Participants

The present study selected the patients who were diagnosed with Small Cell Lung Cancer (SCLC), and administered with irinotecan for the first time.

The inclusion criteria were as follows: (1) age range was 18–70 years; (2) score of Eastern Cooperative Oncology Group (ECOG) assessment was 0–2; (3) estimated survival duration was more than 6 months; (4) function of heart, liver, kidney, or other major organ were normal according to the following laboratory values: cardiac function: left ventricular ejection fraction (LVEF) of cardiac uhrasonography ≥ 50%; blood routine: neutrophils > 1.5 × 10^9^/L; platelets > 100 × 10^9^/L; hemoglobin > 90 g/L; hepatic function: bilirubin < 1.5 × the upper limit of normal (ULN); aspartate aminotransferase (AST)/alanine aminotransferase (ALT) < 2.5 × ULN; renal function: serum creatinine < 1.5 × ULN; endogenous creatinine clearance (CCR) ≥ 60 mL/min (calculated using the Cockcroft-Gault formula); (5) the aims of this study were fully understand and signed, informed consent was provided.

The exclusion criteria were as follows: (1) diarrhea was caused by other reasons (enteritis or inflammatory bowel disease, and medication resulting in diarrhea, such as laxatives, antibiotics), this information was acquired from the records of patients’ medical history; (2) patient was complicated with serious diseases, such as heart, lung, or kidney failure in a decompensated stage; (3) patient was a pregnant or lactating woman; (4) before this study initiation, patient was participating in other clinical trials or finished within the 4 weeks; (5) patients had no ability to understand the study and provide informed consent.

The withdrawal criteria were as follows: (1) severe adverse event (AE) occurred; (2) The irinotecan was replaced by other chemotherapeutic agents for its severe adverse reactions; (3) participants were in contravention of this study’s requirements.

### Sample size calculation, randomization and blinding

This study was designed as a superiority trial with the ratio of 1:1 in the Shengjiang Xiexin decoction (SXD) and control groups. Sample size calculation was performed using PASS software (version 15.0), based on the incidence of diarrhea in preliminary study and literature [21]. And the estimated sample size was approximately 48 each group, with a significance level of 5% (α), a two-sided test, and power of 80% (1-β). A total of 120 subjects were required with a dropout rate of 20%.

An independent external agency specializing in the conduct of clinical trials (Beijing Yin Rui Da Medicine Technology Co. Ltd., Beijing, China) was commissioned to implement randomization, without participation in data management or statistical analysis.

SXD or placebo were sealed in opaque paper boxes labeled by randomization numbers, which were generated using Statistical Analysis System (SAS) software, version 9.4. The participants were assigned a randomization number and received the corresponding package with SXD or the placebo.

All researchers and participants were blinded to the randomization until the trial was completed. Each labeled box had an emergency letter to reveal allocated interventions if a participant had severe adverse events (AEs).

### Uridine diphosphate glucuronosyl transferase 1A1 (UGT1A1) gene testing

UGT1A1 gene testing was performed by an independent biology laboratory (Beijing Ruibo Xingke Biotechnology Co. Ltd., Beijing, China). Each eligible participant provided with at least 2.5 mL of peripheral venous blood for gene testing. UGT1A1*28 genotypes was amplified by polymerase chain reaction (PCR).

### Intervention

According to the National Comprehensive Cancer Network (NCCN) Clinical Practice Guideline for SCLC, all participants received two cycles of chemotherapy as follows: irinotecan 65 mg/m^2^ (body surface area, BSA), cisplatin 30 mg/m^2^ were administered on days 1 and 8, 3 weeks apart.

SXD and placebo were produced by New Green Science and Technology Development Co. Ltd., of Sichuan, China, according to Good Manufacturing Practices (GMP) for Pharmaceutical Products, People’s Republic of China.

The herbal formula of SXD contains eight species of herbs: Shengjiang(Rhizoma Zingiberis Recens), Ganjiang (Rhizoma Zingiberis), Huangqin (Radix Scutellariae), Huanglian (Rhizoma Coptidis), Banxia (Rhizoma Pinelliae), Dangshen (Radix Codonopsis), Dazao (Fructus Jujubae), Gancao (Radix Glycyrrhizae), weighing a total of 66 g, which were made to granules of 6.4 g and packed in aluminum foil bags. The placebo was consisted of maltodextrin, starch, bitters, and food coloring. There was no difference in smell, color, taste, weight and packaging between SXD and placebo. The participants received a dose of 3.2 g (per bag) SXD or placebo twice per day via oral administration, beginning 3 days before each cycle of chemotherapy to 11 days after the start of chemotherapy, the duration of treatments was 14 days in each cycle of chemotherapy, and the total treatment duration was 28 days during the whole trial.

**Fig. 1:**
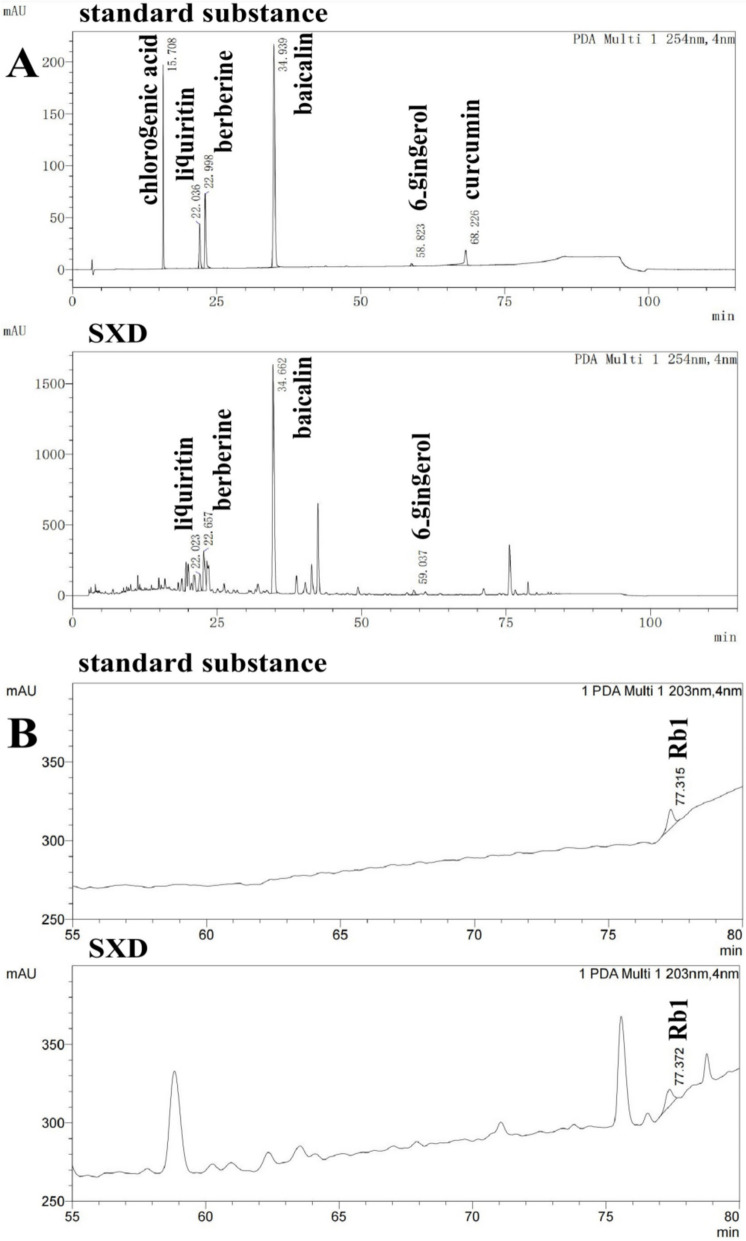
High-performance liquid chromatography (HPLC) analysis of SXD. UV detection at wave length 254 nm for liquiritin, berberine, hydrochloride, baicalin, 6- gingerol (**A**), wave length 203 nm for Ginsenoside Rb1 (**B**); Retention time for liquiritin, berberine, baicalin, 6-gingerol and Ginsenoside Rb1 were 22.02, 22.66, 34.66, 59.04 and 77.37 min respectively. Once the participants developed diarrhea, they were given antidiarrheal medication according to the chemotherapy related diarrhea (CRD) specialist consensus [6]

For high-performance liquid chromatography (HPLC) analysis, a 1 g sample of SXD granules was extracted with water by using an ultrasonic oscillator for 5 min, the extract was then centrifuged at a speed of 10000r/min for 5 min. A 10 ul sample and a 10ul reference substance were directly injected into HPLC system (Shimadzu, Kyoto, Japan). Alphasil VC-C18 chromatographic column (5 μm, 4.6 mm × 250 mm, Acchrom-Tech, China) was used as the stationary phase. The gradient elution was composed of 0.1% formic acid solution and 0.1% carbinol formate at mobile phase. We used a UV detection wavelength of 254 nm for liquiritin, berberine, hydrochloride, baicalin, 6- gingerol; and 203 nm for Ginsenoside Rb1. Retention times for liquiritin, berberine, baicalin, 6-gingerol and Ginsenoside Rb1 were 22.02, 22.66, 34.66, 59.04 and 77.37 min respectively (Fig. 1).

### Outcomes

#### Primary outcome

The primary outcome measure is the incidence of irinotecan-induced diarrhea, as is defined that occurring > 24 h after irinotecan administration and up to the completion of two chemotherapy cycles.

#### Secondary outcomes

Secondary outcomes include the degree of diarrhea and neutropenia, the number of chemotherapy cycles with diarrhea, first occurrence time and duration of diarrhea.

The diarrhea grading criteria was from the Common Terminology Criteria for Adverse Events (CTCAE), Version 5.0, which was released by National Cancer Institute (NCI), for specialized evaluation of adverse reaction caused by chemotherapeutic agents.

Hematologic toxicity is another common adverse reaction related to irinotecan [22]. Therefore, routine blood tests was carried out before and after the day of the chemotherapy infusion. Meanwhile hepatic, renal and blood clotting functions were recorded as safety outcomes. If the outcomes are abnormal obviously, the chemotherapy schedule will be modified as required.

During the trial, any AEs related to SXD administration will be monitored by an independent clinical research associate (CRA), and serious AEs will be reported to the study principal investigator within 24 h, and the necessary diagnosis and treatment will be supplied until the participants recover to a stable condition. In the whole process, the CRA will survey and record in detail.

### Animal experiment

Thirty male Sprague–Dawley rats of SPF grade, weighing 180 ± 20 g, were housed in the animal facility of the Clinical Research Institute of China-Japan Friendship Hospital [SYXK (Beijing) 2016-0043] The rats were divided into three groups (blank group, model group, and SXD group) using the random number table method, with 10 rats in each group.

The irinotecan-induced diarrhea rat model was established following a protocol in the literature [23]. The rats in model group and SXD group were given tail vein injection of irinotecan 150 mg/kg/day for two consecutive days to replicate the irinotecan-induced diarrhea model, while rats in the blank group were initially administered tail vein injection of an equal volume of saline. Then, the rats in SXD group were gavaged with SXD once daily at a dose of 10 g/kg according to their body weight, whereas the rats in the blank and model groups were gavaged with an equal volume of deionized water. All groups were administered SXD or deionized water from 3 days before tail vein injection to the 4th day after the last injection.

All groups of rats were executed and jejunum tissue was collected on the 5th day after the last tail vein injection. The levels of inflammatory factors, including interleukin (IL)-1β, IL-6, and tumor necrosis factor (TNF)-α, and anti-inflammatory factors, including IL-10 and transforming growth factor (TGF)-β1, were measured in the jejunum tissues of rats in each group. The jejunum tissues were homogenized and the supernatants were obtained. Using the ELISA kit, the supernatant was incubated with a horseradish peroxidase (HRP) -labeled antibody, followed by the addition of substrate, and the optical density (OD) of each well was measured at 450 nm using the full wavelength enzyme labeling instrument GO (Thermo Fisher Scientific Inc. USA). The ELISA Calc software was used for data analysis and the results were exported in an Excel spreadsheet.

### Statistical methods

Statistic Package for Social Science (SPSS, v20.0) software was performed for statistical analyses by an independent biostatistician.

The t-test was used to compare the continuous data. The categorical data were analyzed by the chi-square test or Fisher’s exact test was conducted to compare the differences between two groups, such as the incidence of diarrhea. The ranked data were compared between two groups using Wilcoxon rank sum test, such as the grade of diarrhea. And when the continuous data didn’t conform to the normal distribution and homogeneity of variance, we also used the rank sum test for inter-group comparison. P value < 0.05 was considered statistically significant for all tests.

## Result

### Participant enrollment flow

**Fig. 2:**
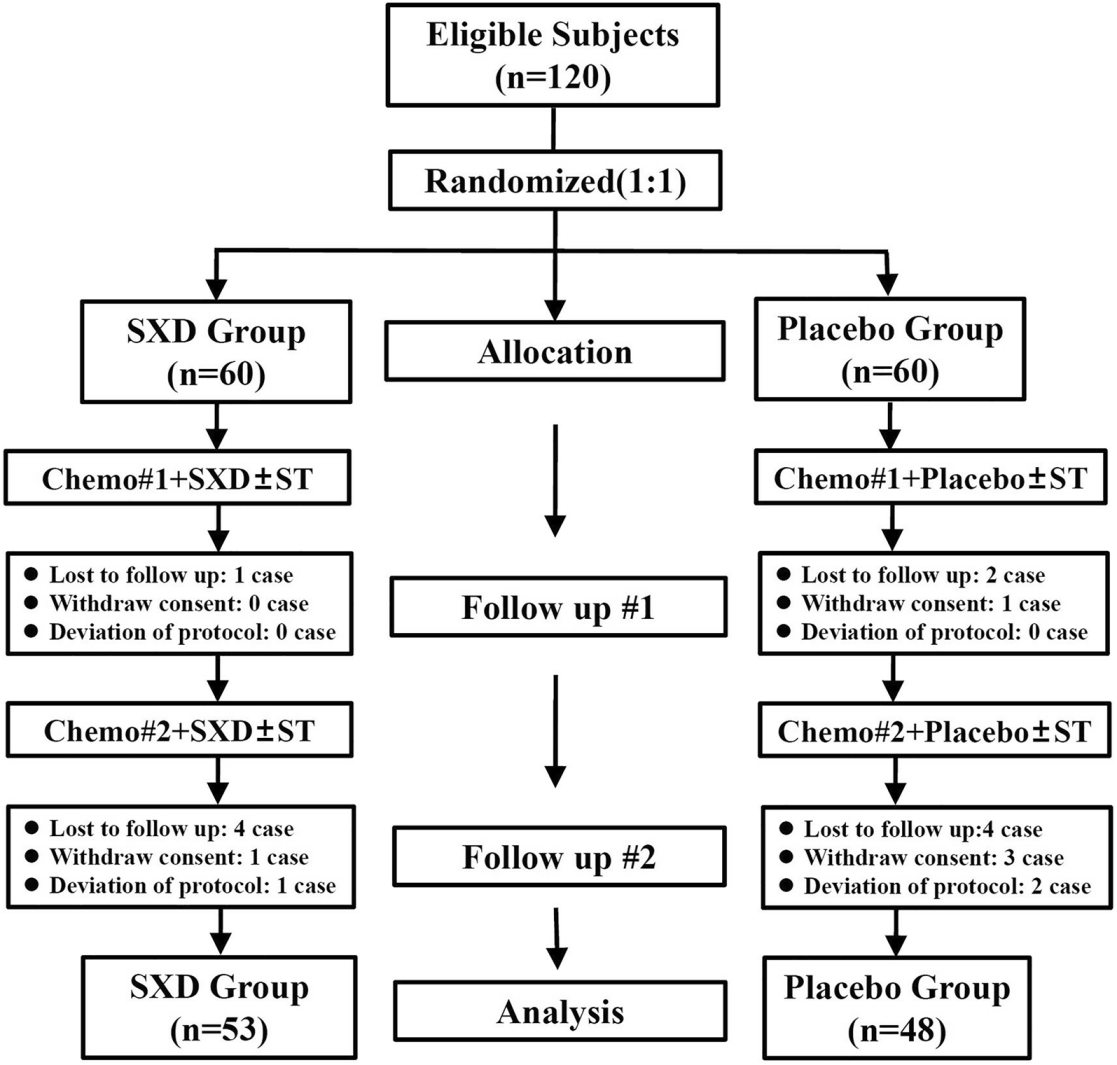
Flow diagram of participant enrollment. *Chemo* chemotherapy, *SXD* Shengjiang Xiexin decoction, *ST* symptomatic treatment

One hundred and twenty eligible subjects were recruited. They were randomly assigned to SXD group or placebo group at ratio of 1:1, and each group had sixty subjects. Seven subjects in SXD group were eliminated due to being lost to follow-up (5 cases), withdrawn consent (1 case), and deviation of protocol (1 case). Twelve subjects were eliminated in placebo group, including being lost to follow-up (6 cases), withdrawn consent (4 cases), and deviation of protocol (2 cases), as shown in Fig. 2.

### Participant characteristics

A total of 101 patients completed the trial, including 53 patients in SXD group and 48 patients in placebo group. There were no significant differences in gender composition, age, BMI, genotype distribution, and cumulative dose of irinotecan between the two groups (all P > 0.05), the baseline data were balanced and comparable, as shown in Table 1.

**Table 1:** The comparison of baseline characteristics of participants

Characteristic	SXD group (n = 53)	Placebo group (n = 48)	P value
Gender			
Female (%)	13 (24.53)	9 (18.75)	0.4823^a^
Male (%)	40 (75.47)	39 (81.25)	
Age (years)			
Mean	73.32	62.1	0.7387^b^
Median	64	63	
BMI (kg/m^2^)			
Mean ± SD	24.24 ± 3.89	23.54 ± 2.95	0.3124^c^
Median	24.34	23.1	
UGT1A1 genotype			
TA6/6 (%)	41(77.36)	36 (75.00)	0.9487^d^
TA6/7 (%)	10 (18.87)	11 (22.92)	
TA7/7 (%)	1 (1.89)	1 (2.08)	
TA6/8 (%)	1 (1.89)	0 (0.00)	
Cumulative dosage of irinotecan (mg/m^2^)			
Mean	228.7	214.04	0.8773^b^
Median	227.27	221.61	

^a^Chi-square test

^b^Wilcoxon rank sum test

^c^t -test

^d^Fisher’s exact test

### Outcomes

The incidence of diarrhea in SXD group and the control group was 26.42% and 52.08%, respectively (P = 0.0081). There were 8 cases of grade 1 diarrhea, 3 cases of grade 2 diarrhea and 3 cases of grade 3 diarrhea in SXD group, and 11 cases of grade 1 diarrhea, 13 cases of grade 2 diarrhea and 1 case of grade 3 diarrhea in the control group. There was a statistically significant difference in the degree of diarrhea between the two groups (P = 0.0097). There were 6 cases using antidiarrheal drugs in the TCM group and 19 cases in the control group, and the use rate of antidiarrheal drugs was 11.32% and 39.58%, respectively (P = 0.001) (Table 2).

**Table 2:** The comparison of the outcomes between two groups

	SXD group (n = 53)	Placebo group (n = 48)	P value
Diarrhea			
Yes	14 (26.42)	25 (52.08)	0.0081^a^
No	39 (73.58)	23 (47.92)	
No. of chemo cycles with diarrhea			
0 (%)	39 (73.58)	23 (47.92)	0.0278^a^
1 (%)	8 (15.09)	16 (33.33)	
2 (%)	6 (11.32)	9 (18.75)	
Degree of diarrhea			
0 (%)	39 (73.58)	23 (47.92)	0.0097^b^
1 (%)	8 (15.09)	11 (22.92)	
2 (%)	3 (5.66)	13 (27.08)	
3 (%)	3 (5.66)	1 (2.08)	
4 (%)	0 (0.00)	0 (0.00)	
5 (%)	0 (0.00)	0 (0.00)	
Antidiarrheal medication			
Yes (%)	6 (11.32)	19 (39.58)	0.0010^a^
No (%)	47 (88.68)	29 (60.42)	
First occurrence time of diarrhea (day)			
Mean	5.29	3.48	0.1615^b^
Median	6	3	
Duration of diarrhea (day)			
Mean	7.83	11.11	0.5909^b^
Median	7	7	
Degree of neutropenia			
0 (%)	30 (56.60)	19 (39.58)	0.0465^b^
1 (%)	11 (20.75)	10 (20.83)	
2 (%)	7 (13.21)	9 (18.75)	
3 (%)	5 (9.43)	10 (20.83)	
4 (%)	0 (0.00)	0 (0.00)	

^a^Chi-square test

^b^Wilcoxon rank sum test

However, there was no statistically significant difference in the time to first onset and duration of diarrhea between the two groups. In addition, the incidence of grade 1–3 neutropenia in the two groups was 20.75% vs 20.83%, 13.21% vs 18.57%, and 9.43% vs 20.83%, respectively, there was a statistically significant difference in the grade distribution of neutropenia between the two groups (P = 0.0465) (Table 2).

The stratified analysis was implemented according to the genotype of UGT1A1. The cases in two groups were divided into the stratification of low risk (wild type) and high risk (heterozygous mutation and homozygous mutation) of UGT1A1.

In the low- risk stratification, there were 41 cases in the SXD group and 36 cases in the control group, and the incidence of diarrhea was 29.27% and 47.22%, respectively. However, there was no statistically significant difference between two groups (P = 1.1047). Diarrhea grade distribution of the two groups was shown in Table 3, and there was no statistically significant difference between the two groups (P = 0.2104). There were 5 cases using antidiarrheal drugs in SXD group and 12 cases in the control group, and the use rate of antidiarrheal drugs was 12.2% and 33.3%, respectively (P = 0.0257) (Table 3).

**Table 3:** The comparison of the outcomes between two groups with low-risk gene

	SXD group (n = 41)	Placebo group (n = 36)	P value
Diarrhea			
Yes	12 (29.27)	17 (47.22)	0.1047^a^
No	29 (70.73)	19 (52.78)	
Degree of diarrhea			
0 (%)	29 (70.73)	19 (52.78)	0.2104^b^
1 (%)	7 (17.07)	8 (22.22)	
2 (%)	3 (7.32)	8 (22.22)	
3 (%)	2 (4.88)	1 (2.78)	
4 (%)	0 (0.00)	0 (0.00)	
5 (%)	0 (0.00)	0 (0.00)	
Antidiarrheal medication			
Yes (%)	5 (12.20)	12 (33.33)	0.0257^a^
No (%)	36 (87.80)	24 (66.67)	

^a^Chi-square test

^b^Fisher’s exact test

In the high-risk stratification, there were 11 cases in SXD group and 12 cases in the control group, and the incidence of diarrhea was 9.09% and 66.67%, respectively, the difference was statistically significant (P = 0.0094). There were 1 case of grade 1 diarrhea in SXD group, 3 cases of grade 1 and 5 cases of grade 2 diarrhea in the control group. There was a statistically significant difference in the degree of diarrhea between the two groups (P = 0.0105). One patient in SXD group and 7 patients in the control group used antidiarrheal drugs, and the use rate of antidiarrheal drugs was 8.33% and 58.33%, respectively (P = 0.0272) (Table 4).

**Table 4:** The comparison of the outcomes between two groups with high-risk gene

	SXD group (n = 12)	Placebo group (n = 12)	P value
Diarrhea			
Yes	2 (16.67)	8 (66.67)	0.0361^a^
No	10 (83.33)	4 (33.33)	
Degree of diarrhea			
0 (%)	10 (83.33)	4 (33.33)	0.0105^a^
1 (%)	1 (8.33)	3 (5.00)	
2 (%)	0 (0.00)	5 (41.67)	
3 (%)	1 (8.33)	0 (0.00)	
4 (%)	0 (0.00)	0 (0.00)	
5 (%)	0 (0.00)	0 (0.00)	
Antidiarrheal medication			
Yes (%)	1(8.33)	7 (58.33)	0.0272^a^
No (%)	11(91.67)	5 (41.67)	

^a^Fisher’s exact test

### Adverse events

In this clinical trial, there were no obvious adverse events related to decoction in SXD group and the control group. Only two cases appearred mild abdomen distension and constipation in SXD group. There were no significant changes in liver and kidney function and coagulation function before and after treatment in the two groups (Table 5).

**Table 5:** The comparison of safety indicators before and after treatment in two groups

Group/indicator	Baseline	Chemo#1	P value	Chemo#2	P value
SXD group					
ALT(IU/L)	28.34 ± 23.28	28.68 ± 41.93	0.431^a^	25.55 ± 18.73	0.6669^a^
AST(IU/L)	24.55 ± 14.74	21.34 ± 9.83	0.0133^a^	20.91 ± 7.95	0.1047^a^
Urea(mmol/L)	4.89 ± 1.63	5.49 ± 2.93	0.4248^a^	5.21 ± 1.99	0.3835^a^
CR(μmol/L)	63.73 ± 14.69	65.32 ± 17.12	0.1252^a^	62.58 ± 15.04	0.5677^a^
D-D(mg/L)	0.83 ± 0.97	0.91 ± 1.08	0.0063^a^	0.84 ± 1.06	0.9945^a^
Placebo group					
ALT(IU/L)	22.95 ± 19.31	22.07 ± 20.32	0.9227^a^	20.30 ± 16.06	0.8679^a^
AST(IU/L)	21.66 ± 10.63	20.28 ± 9.25	0.3717^a^	19.69 ± 10.03	0.2507^a^
Urea(mmol/L)	10.82 ± 41.95	5.30 ± 1.76	0.6439^a^	5.49 ± 2.74	0.4867^a^
CR(μmol/L)	64.32 ± 12.81	64.12 ± 14.35	0.8639^b^	67.24 ± 21.45	0.8027^a^
D-D(mg/L)	1.25 ± 1.53	1.26 ± 1.42	0.8298^a^	1.39 ± 3.21	0.4952^a^

^a^Wilcoxon rank sum test

^b^t -test

### SXD can reduce the level of jejunal inflammation

**Fig. 3:**
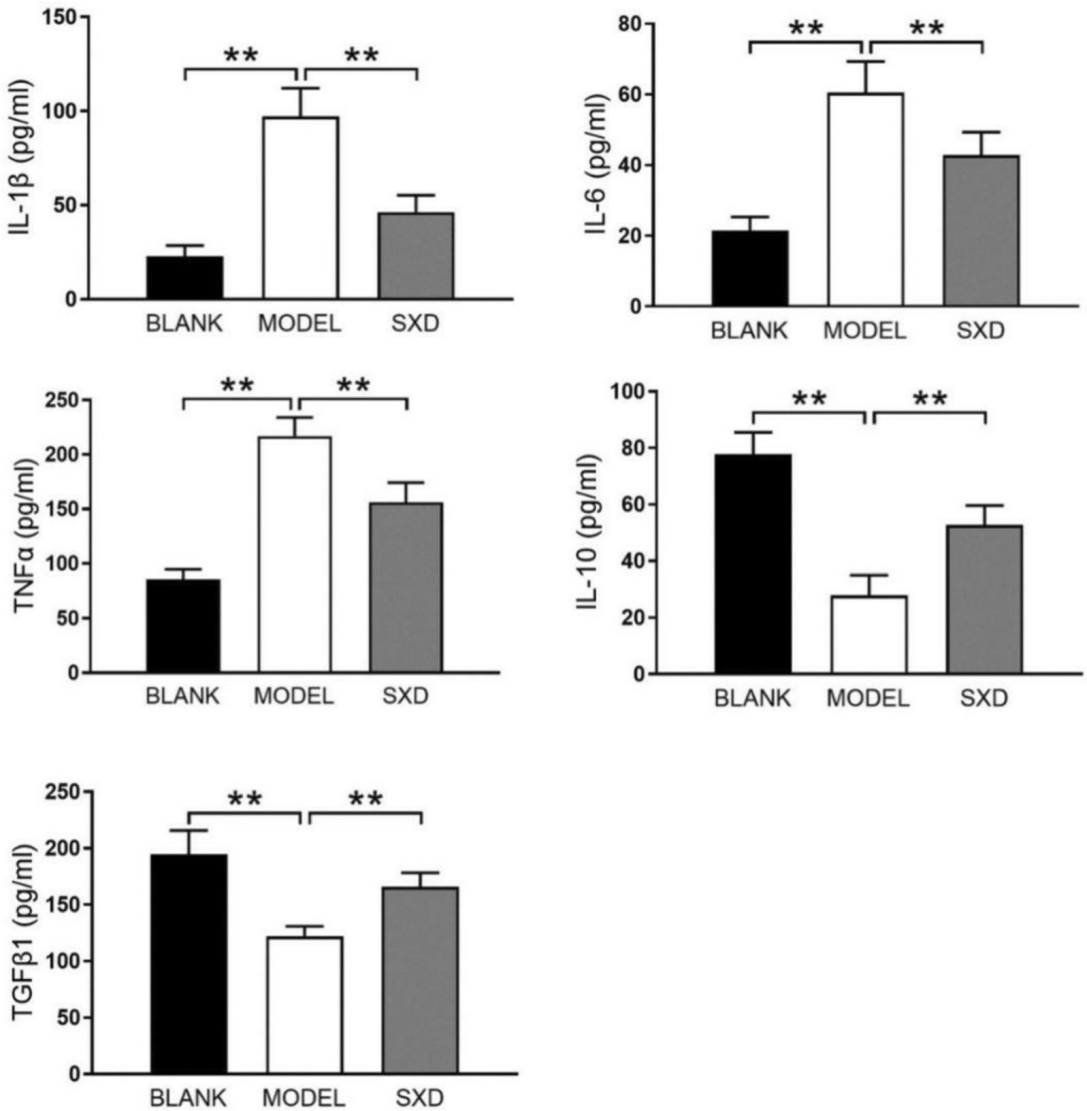
Expression of inflammatory factors IL-1β, IL-6, TNF-α/anti- inflammatory factor IL-10, TGF-β1 in rat jejunum. **P < 0.01

Compared with the blank group, the inflammatory factors (IL-1β, IL-6 and TNF-α) were significantly higher (P < 0.01) and the anti-inflammatory factors (IL-10 and TGF- β1) were significantly lower (P < 0.01) in the model group. In contrast, the levels of inflammatory factors in SXD group were significantly lower than those in model group (P < 0.01), while anti-inflammatory factors were significantly higher (P < 0.01) (Fig. 3). This result indicated that SXD plays a protective role in the intestinal mucosa by inhibiting intestinal inflammation, thus reduces the occurrence of diarrhea.

## Discussion

Irinotecan-induced diarrhea resulted in chemotherapy interruption or cessation, and even occasionally with life-threatening dehydration and electrolyte disruption. All above severely affected the prognosis and quality of life of the cancer patients. The clinical strategies were difficult to decide due to the limited therapeutic efficacy and absence of prophylaxis in western medicine.

In recent years, more and more studies on potential efficacy of TCM prescriptions in irinotecan-induced diarrhea, had the chance to change the current understanding and address knowledge gaps. However, the samples of these studies are generally small, and the UGT1A1 gene status of the subjects is not detected, which may lead to biased results. Therefore, Our study was designed with a multi-center, large sample, randomized controlled trial, and added with the detection of the gene status of the subjects, stratified analysis of the results was performed to fill in the gaps of previous studies.

In this study, the early intervention with SXD significantly reduced the incidence and grade of diarrhea in patients receiving irinotecan chemotherapy, this result was helpful to enhance the physical strength and increase the tolerance to chemotherapy for patients. Meanwhile, SXD also reduced the use of antidiarrheal drugs, and showed some efficacy in the first occurrence time and duration of diarrhea. In addition to diarrhea, SXD decreased the degree of neutropenia as well. All of these lowered additional medical expense.

It is well known that UGT1A1 polymorphism has a significant relationship with irinotecan-induced gastrointestinal toxicity, UGT1A1 homo/heterozygous mutant status was associated with severe diarrhea and mucositis [24]. Thus, current available strategy towared diarrhea was dose reduction according to UGT1A1 genotype, however, in a prospective multi-centre study, dosing of irinotecan was guided by UGT1A1 genotype, homozygous variant carriers (with high-risk gene) received an 30% dose reduction of irinotecan, the incidence of febrile neutropenia and grade ≥ 3 diarrhea were not significantly reduced [25]. Another study revealed a similar result, in the population with high-risk UGT1A1 genotype, the dose reduction not only lowered the efficacy of chemotherapy, but also did not significantly reduce the occurrence of adverse reactions [26]. Thus, dose reduction was not a better choice to lower the risk of diarrhea in clinical practice.

Our previous study had shown that SXD could reduce the risk of diarrhea in UGT1A1 mutation patients accepting standard dose irinotecan. Through intervention of SXD in advance, the incidence of diarrhea in patients with UGT1A1 mutation was nearly equal to that of patients with the wild-type [18].

In this study, we further explored prophylactic effect of SXD based on UGT1A1 gene status by stratified analysis. For the low-risk population, SXD group showed a lower incidence of diarrhea than placebo group, but the difference was not statistically significant, which may be related to the relatively low risk of chemotherapy-induced diarrhea in this population. For the high-risk population, SXD showed more obvious efficacy, which was consistent with the conclusion of our previous study.

Besides, in SCLC patients, the application dosage of irinotecan was significantly lower than gastrointestinal cancer patients. And a meta-analysis revealed, the association between UGT1A1 and irinotecan-induced toxicity in low-dose irinotecan is stronger than higher doses [27].

To sum up, the result of our study filled the limitation of dose reduction in high-risk population, especially for SCLC patients with relative lower dose. SXD enriched the choice of intervention strategies.

Irinotecan-induced diarrhea is related to intestinal inflammatory damage, and the inflammatory response will further increase the concentration of irinotecan and its toxic metabolite SN-38, thus further aggravating intestinal toxicity and forming a vicious cycle [28, 29]. Therefore, on the basis of evaluating the clinical efficacy of SXD, our study conducted a preliminary exploration of its mechanism of action, and the results showed that SXD alleviated inflammation-related intestinal damage by changing the expression levels of intestinal inflammatory and anti-inflammatory factors, which was closely related to the anti-inflammatory effects of baicalin and berberine, as the main components of SXD [30, 31], and this mechanism was consistent with previous research [32]. As far as the upstream pathway of regulating inflammatory response by SXD still needs further research.

Our study also had several limitations, it only observed two cycles of chemotherapy, the evaluation of the efficacy of chemotherapy was not sufficient, and more and sufficient clinical evidence is still needed to confirm whether adjuvant therapy with Chinese herbal medicine during chemotherapy alters the chemotherapy response. This study used the CTCAE diarrhea grading system to evaluate CRD as previous studies. However, this system only focuses on the frequency of diarrhea, not included concomitant symptoms such as abdominal pain, distension, and cramps, it cannot provide a complete assessment of CRD.

## Conclusions

SXD has a significant preventive effect on irinotecan-induced diarrhea in high-risk population, enhances the tolerance to irinotecan chemotherapy for high-risk population, and promotes the completion of chemotherapy plan. The mechanism of action of SXD was related to the regulation of inflammatory factors.

## Supplementary Information

Additional file 1. CONSORT 2010 Checklist

## Data Availability

The data sources used in this study are available from the corresponding author on reasonable request.
